# Hematological and biochemical parameters correlated to hemorheology in Canine Monocytic Ehrlichiosis

**DOI:** 10.1590/S1984-29612024076

**Published:** 2024-12-20

**Authors:** Saulo Pereira Cardoso, Adenilda Cristina Honorio-França, Luana Paula Sales Silva, Maria Clara Bianchini Neves, Arlyson Sousa Ferreira, Arleana do Bom Parto Ferreira Almeida, Eduardo Luzía França, Luciano Nakazato, Valéria Régia Franco Sousa

**Affiliations:** 1 Unidade de Ensino e Produção Agropecuária, Instituto Federal de Educação, Ciência e Tecnologia de Mato Grosso – IFMT, Barra do Garças, MT, Brasil; 2 Programa de Pós-graduação em Ciências Veterinárias, Faculdade de Medicina Veterinária – FAVET, Universidade Federal de Mato Grosso – UFMT Cuiabá, MT, Brasil; 3 Laboratório de Imunologia, Instituto de Ciências Biológicas e da Saúde, Universidade Federal de Mato Grosso – UFMT, Campus Universitário do Araguaia II, Barra do Garças, MT, Brasil

**Keywords:** Ehrlichia canis, blood viscosity, hysteresis curve, anemia, acute phase protein, Ehrlichia canis, viscosidade sanguínea, curva de histerese, anemia, proteínas de fase aguda

## Abstract

Canine monocytic ehrlichiosis (CME) is an infectious disease that causes hematological changes in dogs. This study investigated the correlations between hematological and hemorheological parameters, serum proteins, and triglycerides in dogs with CME. Fifty-nine blood and/or bone marrow samples were collected from dogs with or without clinical signs of CME. Blood samples preserved with EDTA were subjected to rheological analysis to investigate blood viscosity. Fourteen dogs with *Ehrlichia canis* infection (CME group) and 20 without clinical signs and *E. canis* infection (CG) were selected by qPCR based on Ecaj_0503 gene. The blood viscosity of the infected dogs (CMEG) was lower than that of the control group (CG). The mean values and standard error of erythrocytes (CG: 6.71 ± 0.20; CMEG: 4.82 ± 0.23), platelets (CG: 235.6 ± 15.67; CMEG: 151.07 ± 16.51), and albumin (CG: 3.04 ± 0.15; CMEG: 2.65 ± 0.12) in the infected dogs were lower (p<0.005) than those in the control group. The decrease in erythrocytes influenced the decrease in blood viscosity. Total protein, albumin and triglycerides levels correlated with blood viscosity in infected dogs. Overall, this study shows that dogs with CME have decreased blood viscosity primarily due to anemia and interactions with negative acute-phase proteins.

## Resumo

A erliquiose monocítica canina (EMC) é uma doença infecciosa que causa alterações hematológicas em cães. Este estudo investigou as correlações entre os parâmetros hematológicos e hemorreológicos, as proteínas séricas e os triglicerídeos em cães com EMC. Cinquenta e nove amostras de sangue e/ou medula óssea foram coletadas de cães com ou sem sinais clínicos de EMC. As amostras de sangue preservadas com EDTA foram submetidas à análise reológica para investigar a viscosidade do sangue. Quatorze cães com infeção por *Ehrlichia canis* (CMEG) e 20 sem sinais clínicos e infecção por *E*. *canis* (CG) foram selecionados por qPCR com base no gene Ecaj_0503. A viscosidade do sangue dos cães infectados (CMEG) foi inferior à do grupo de controle (GC). Os valores médios e o erro padrão dos eritrócitos (GC: 6,71 ± 0,20; CMEG: 4,82 ± 0,23), plaquetas (GC: 235,6 ± 15,67; CMEG: 151,07 ± 16,51) e albumina (GC: 3,04 ± 0,15; CMEG: 2,65 ± 0,12) nos cães infectados foram inferiores (p<0,005) aos do grupo de controle. A diminuição dos eritrócitos influenciou a diminuição da viscosidade do sangue. Os níveis de proteínas totais, albumina e triglicerídeos correlacionaram-se com a viscosidade do sangue nos cães infectados. Em termos gerais, este estudo mostra que os cães com EMC apresentam uma diminuição da viscosidade sanguínea principalmente devido à anemia e a interações com proteínas negativas da fase aguda.

## Introduction

Canine monocytic Ehrlichiosis (CME) is a vector-borne disease affecting dogs in tropical and subtropical regions (Sainz et al., 2015). *Ehrlichia canis* is a bacterium of the phylum Proteobacteriota, class Alphaproteobacteria, and order Rickettisiales (Schoch et al., 2020), which infects the phagocytic mononuclear system, with a preference for macrophages. The main vector of *E. canis* is *Rhipicephalus sanguineus* sensu lato (Acari: Ixodida), which transmits bacteria through blood meals. Nymphs and adult ticks can transmit the pathogen to other hosts upon infection (Aziz et al., 2022).

The clinical signs are related to the pathogenesis of CME, which is systemic and affects multiple dog tissues, causing vascular lesions (Castro et al., 2022). The differentiation of the three phases of the disease (acute, subclinical, and chronic) in natural infections by *E. canis* needs to be clarified, as clinical and laboratory signs are non-specific and common to different phases (Diniz & Aguiar, 2022). Laboratory findings, such as anemia and thrombocytopenia, are common in dogs with CME, particularly in the acute phase (Aziz et al., 2022). The acute phase is characterized by fever, hemorrhage, lymphadenopathy, anorexia, depression, anemia, and thrombocytopenia. The subclinical phase usually presents thrombocytopenia and hyperglobulinemia. Dogs can develop chronic form, which has a worse prognosis and is characterized by pancytopenia, bone marrow aplasia, hemorrhage, and secondary infections (Diniz & Aguiar, 2022).

CME can be diagnosed using various techniques, including the visualization of *Ehrlichia* morulae within monocytes in blood smears, rapid serological tests using qualitative commercial kits, enzyme-linked immunosorbent assay (ELISA), indirect immunofluorescence, and molecular polymerase chain reaction techniques (conventional PCR or real-time PCR) (Sainz et al., 2015).

Hematological changes caused by infection with bacteria of the Anaplasmataceae family, such as anemia and thrombocytopenia, may result in changes in blood rheological parameters. As such, dogs infected with Anaplasmataceae agents show a decrease in blood viscosity compared to healthy dogs (Cardoso et al., 2020). In dogs infected with *Leishmania infantum*, the decrease in erythrocytes caused by this disease is also related to a decrease in blood viscosity and an increase in shear rate (Silva et al., 2018).

Hemorheological behavior, a dynamic phenomenon, is influenced by blood viscosity and several other factors, such as circulating blood volume, the number of cells, components present in plasma, peripheral resistance of blood vessels, and intravascular blood flow pressure (Martins e Silva, 1983; Silva et al., 2018). Other parameters that can alter the rheological behavior of blood include circulating lipids, which affect the viscosity of blood plasma (Irace et al., 2014), and immunoglobulins, which can directly or indirectly influence blood viscosity by increasing erythrocyte aggregation (Kwaan, 2010).

The increased concentration of blood components, such as leukocytes and platelets, in the blood can further disrupt the normal flow of erythrocytes, primarily in the microcirculation, owing to the smaller caliber of the capillaries. Viscosity is altered if the illness alters the number of cells, erythrocyte deformability, or serum components (Martins e Silva, 1983; Baskurt & Meiselman, 2003). Rheometry is an auxiliary, low-cost technique for monitoring the hematological conditions and hemorheological behavior of animals with infectious diseases. However, few studies in veterinary medicine have been conducted in this field, and a wide range of research fields still require investigation.

This study characterized the hemorheological profile of dogs infected with *E. canis* and correlated it with the hematological, serum proteins, and triglycerides.

## Material and methods

### Animals

In the years 2021 and 2022, blood and/or bone marrow samples were randomly collected from 59 dogs at a private veterinary hospital in the city of Barra do Garças, Mato Grosso, Brazil (-15.8891, 15° 53' 24” S, -52.2634, 52° 15' 24” W). Samples that tested positive for *E. canis* using qPCR were included in this study. In addition, samples showing DNA amplification for *L. infantum, Babesia* spp., *Hepatozoon* spp. and *Anaplasma platys*, were excluded. Nineteen dogs that tested positive for *Leishmania* and six for *Anaplasma platys* were excluded. None tested positive for *Babesia* spp. or *Hepatozoon* spp.

Two groups were formed: 14 dogs with CME and 20 dogs without clinical signs and DNA amplification of *E. canis* (control group). Blood and serum samples were obtained from each dog for hemorheological, hematological, and biochemical evaluations. Further, qualitative and semi-quantitative data, including sex, age, body score, and clinical signs (anorexia, fever, lymphadenopathy, splenomegaly, pale mucosa, and bleeding) were recorded.

### Qualitative Real-Time PCR (qPCR) for *E. canis* and *L. infantum*

For molecular analysis, DNA was extracted from whole blood and/or bone marrow using the phenol-chloroform method followed by isopropanol precipitation (Sambrook & Russel, 2001). The DNA was dissolved in 50µl of ultrapure water, and its concentration was measured using a NanoDrop™ 2000/2000c Spectrophotometer (Thermo Scientific). All samples were treated with RNAse. Conventional PCR for the endogenous canine β-globin gene was performed (Quaresma et al., 2009). The *E. canis* DNA detection process used the primers E_can0503F (5'-CAG CAA ATT CCA ATC TGC ACT TC-3') and E_can0503R (5'-GAG CTT CCA ATT GAT GGGTCT G-3') in which the gene Ecaj_0503 encodes 147 bp of a hypothetical protein [system E_can0701] (Socolovschi et al., 2012).

The qPCR analysis was performed in triplicate using a StepOne™ Real-Time PCR System Sequence Detection (Thermo Fisher Scientific, Massachusetts, USA). Reactions were prepared in a final volume of 25µl containing non-specific double-stranded DNA intercalators (Sybr Green® Master Mix, Thermo Fisher Scientific, Massachusetts, USA), 0.3µM of each primer and 2µl of target DNA. The reaction consisted of 94°C for 10 minutes, 40 cycles of 94°C for 15 seconds, and 60°C for 30 seconds for extension. Additionally, a standard curve was established for each assay using a known amount of the TOPO PCR 2.1 plasmid (Invitrogen Corp.) containing the *E. canis* DNA in a serial dilution of 12x10^7^ to 12x10^1^. The reaction showed efficiency of 96% (R2= 0,997 ε = 96,257 and Slope= - 3,415) with melting temperature of 77, 06° C. In all reactions was used a negative control containing DNA-free water and the positive control sample was tested for *E. canis* by nested PCR (Makino et al., 2016).

For *L. infantum* qPCR, the primers RV1-5'-CTT TTC TGG TCC GGG TAG G-3' and RV2-5'-CCA CCT GGC TAT TTT ACA CCA-3' were used, which amplify 145 bp of the *L. infantum* kinetoplast (Lachaud et al., 2002). Succinctly, reactions were prepared in a final volume of 25 μL containing 2 μL of target DNA, SYBR Green Master Mix, and 0.3 μM of each primer. The reaction was: an initial incubation step at 94 °C for 10 min, 40 cycles of amplification at 94 °C for 15 s and 60 °C for 60 s. A standard curve was established for each assay using known amounts of TOPO PCR 2.1 plasmid (Invitrogen Corp.) containing the *L. infantum* kDNA gene. The reaction showed efficiency of 99% (R2= 0,972 ε = 99,548 and Slope= - 3,333) with melting temperature of 84,06° C. In each assay, a negative control containing DNA-free water was used (Ayres et al., 2022).

### Conventional and Nested Polymerase Chain Reaction (PCR) for *Babesia* spp., *Rangelia vitalli, Hepatozoon* spp., and *A. platys*

In the PCR for Piroplasmida (*Babesia* spp. and *Rangelia vitalli)* and *Hepatozoon* spp. primers BAB143-167 -5'- CCG TGC TAA TTG TAG GGC TAA TAC A - 3' and BAB694-667 - 5'- GCT TGA AAC ACT CTA RTT TTC TCA AAG - 3' were used, which amplifies a region of approximately 550 bp of the 18S rRNA gene (Almeida et al., 2012; Wolf et al., 2016). The amplified products were fractionated by electrophoresis on a 1.5% agarose gel, stained with Gel Red, and visualized using a transilluminator (UV-300 nm). Nested PCR was performed (Platys-F: AAGTCGAACGGATTTTTGTC Platys-R: CTTTAACTTACCGAACC) to exclude infection by *A. platys* (Inokuma et al., 2001). In all reactions was used a negative control containing DNA-free water and an appropriate positive control for each agent, i.e. *Babesia* spp. (Castro et al., 2020), *Hepatozoon* spp. (Maia et al., 2019) and *A. platys* (Cruz et al., 2017).

### Hemorheological parameters

The hemorheological parameter was obtained using the compact modular rheometer, model Anton-Paar® Cone-Plate—MCR 102 [Anton Paar® GmbH, Ostfildern, Germany]. The graphs were obtained with the Rheoplus® software, as proposed by França et al. (2014). Succinctly, 750μL of whole blood with EDTA was placed in the rheometer at a temperature of 37ºC with 60 flow measurement points to obtain the blood viscosity.

### Hematological and biochemical analyses

According to Sirois (2020), for hematological analysis, samples of whole blood with EDTA were processed on an automated Icounter Vet device, model D Check D Plus [DIAGNO®, Belo Horizonte, Brazil]. Serum samples from the dogs were sent for biochemical analysis to determine the values of total protein, albumin, globulins, and triglycerides in an automated Smart 200+ device [BioTécnica®, Varginha, Brazil]. The reference values proposed by Rizzi et al. (2010) were used.

### Statistical analysis

Data were expressed as mean ± standard error (SE). Statistical analyses were performed with the BioEstat® version 5.0 software [Mamirauá Institute, Belém, Brazil]. A D'Agostino normality test and t de Student were used to analyze independent variables data statistically. In addition, the Pearson's linear correlation was used. Results were considered significant when the P-value was less than 0.05 (p< 0.05).

## Results

Table 1 summarizes data from the dogs selected in both groups, such as sex, age, and body score. Dogs with *E. canis* infection presented with various clinical signs in their history, which were obtained from medical records. Changes in blood count and biochemical test results were also assessed (Table 1).

**Table 1:** The number of dogs for each of the variables: sex, age, and body score (from 1 to 5 scale).

**Group**	**Sex**			**Years**			**Body score**	
	**F/M**	**n**		**Class**	**n**		**Scale**	**n**
Control	F	10		1 a 5	16		< 3	0
	M	10		6 a 10	2		3	18
				>10	2		> 3	2
				NI	0		NI	0
	subtotal	20		subtotal	20		subtotal	20
*E. canis* (+)	F	10		1 a 5	9		< 3	1
	M	4		6 a 10	4		3	10
				>10	1		> 3	2
				NI	0		NI	1
	subtotal	14		subtotal	14		subtotal	14
Total		34			34			34

**Caption**: F = female; M = male; n = number of dogs; NI = not informed; (+) positive result for *E. canis*.

Regarding hematological parameters (Table 2), significant differences in erythrocytes (CG: 6.71 ± 0.20 10^6^/mm^3^; CMEG: 4.82 ± 0.23 10^6^/mm^3^), hematocrit (CG: 45.23 ± 1.39%; CMEG: 32.17 ± 1.56%), hemoglobin (CG: 15.76 ± 0.48 g/dL; CMEG: 10.55 ± 0,65 g/dL), platelets (CG: 235.6 ± 15.67 10^6^/mm^3^; CMEG: 151.07 ± 16.51 10^6^/mm^3^), were found between the two groups, with the CME group showing decreased concentrations of each of these mentioned parameters compared to controls. As shown in Table 2, dogs with CME (2.65 ± 0.12 g/dL) showed a statistically significant (p < 0.05) decrease in albumin levels compared to the control group.

**Table 2:** Hematological and biochemical parameters of dogs positive for *E. canis* and controls expressed as mean values and standard error (Student’s t-test).

**Hematologic parameters**	**Control**	***E. canis* (+)**	**P**
Erythrocytes (10^6^/mm^3^)	6.71 ± 0.20	4.82 ± 0.23	< 0.05
Hematocrit (%)	45.23 ± 1.39	32.17 ± 1.56	< 0.05
Hemoglobin (g/dL)	15.76 ± 20.48	10.55 ± 0.65	< 0.05
Platelets (10^3^/mm^3^)	235.6 ± 15.67	151.07 ± 16.51	< 0.05
Leukocytes (10^3^/mm^3^)	10.28 ± 0.69	8.78 ± 0.88	0.17
Neutrophils (10^3^/mm^3^)	6.55 ± 0.46	5.87 ± 0.65	0.11
Lymphocytes (10^3^/mm^3^)	1.64 ± 0.26	1.68 ± 0.27	0.47
Monocytes (10^3^/mm^3^)	0.31 ± 0.02	0.86 ± 0.22	0.43
Eosinophils (10^3^/mm^3^)	0.41 ± 0.06	0.44 ± 0.09	0.30
**Biochemical parameters**			
Total protein (g/dL)	7.20 ± 0.13	7.85 ± 0.25	0.10
Albumin (g/dL)	3.04 ± 0.15	2.65 ± 0.12	< 0.05
Globulins (g/dL)	4.15 ± 0.19	5.13 ± 0.27	< 0.05
Triglycerides (mg/dL)	80.06 ± 5.22	75.70 ± 6.85	0.36

**Caption**: (+) positive result for *E. canis,* P values ≤ 0.05 were considered statistically significant.

In dogs with CME, serum albumin showed a linear correlation of medium strength with erythrocytes (r = 0.55; p < 0.05), hematocrit (r = 0.56; p < 0.05), hemoglobin (r = 0.57; p < 0.05), and platelets (r = 0.62; p < 0.05) (Table 3). In the control group, only a direct proportional average correlation was observed between albumin and platelet count (r = 0.53; p < 0.05). Medium-strength and directly proportional correlations were also observed between triglycerides and erythrocytes (r = 0.45; p < 0.05), as well as between triglycerides and neutrophils (r = 0.50; p < 0.05).

**Table 3:** Pearson's linear correlation between the hematological and biochemical parameters of dogs positive for *E. canis* and controls.

**Hematologic parameter**	**Total proteins**				**Albumin**				**Globulin**				**Triglycerides**			
	**Control**		***E. canis* (+)**		**Control**		***E. canis* (+)**		**Control**		***E. canis* (+)**		**Control**		***E. canis* (+)**	
	**r**	**P**	**r**	**P**	**r**	**P**	**r**	**P**	**r**	**P**	**r**	**P**	**r**	**P**	**r**	**P**
**Erythrocytes**	-0.03	0.89	0.23	0.41	-0.15	0.54	0.55	< 0.05	0.10	0.69	-0.02	0.93	-0.08	0.73	0.53	< 0.05
**Hematocrit**	-0.02	0.92	0.18	0.52	-0.19	0.42	0.56	< 0.05	0.14	0.57	-0.07	0.79	-0.01	0.96	0.48	0.07
**Hemoglobin**	0.04	0.87	0.25	0.37	-0.23	0.33	0.57	< 0.05	0.21	0.37	-0.01	0.96	-0.10	0.96	0.42	0.12
**Platelets**	-0.02	0.94	-0.11	0.68	0.53	< 0.05	0.62	< 0.05	-0.33	0.27	-0.38	0.17	0.16	0.49	-0.08	0.78
**Leukocytes**	0.01	0.99	0.60	<0.05	0.08	0.74	0.22	0.44	-0.07	0.79	-0.02	0.46	0.09	0.57	0.34	0.27
**Neutrophils**	0.01	0.99	0.43	0.11	0.16	0.51	0.36	0.19	-0.13	0.60	0.25	0.38	0.24	0.32	0.65	< 0.05
**Lymphocytes**	-0.05	0.83	0.18	0.53	-0.44	0.08	-0.22	0.44	0.31	0.24	0.26	0.35	-0.28	0.29	-0.31	0.26
**Monocytes**	0.22	0.39	0.65	<0.05	-0.03	0.91	0.05	0.84	0.16	0.50	0.58	<0.05	-0.19	0.44	-0.01	0.95
**Eosinophils**	-0.46	0.06	0.49	0.07	-0.10	0.70	0.22	0.43	-0.35	0.18	0.36	0.20	-0.02	0.92	-0.14	0.12

**Caption**: (+) positive result for *E. canis.,* coefficient of correlation (r), P values ≤ 0.05 were considered statistically significant.

**Figure 1:**
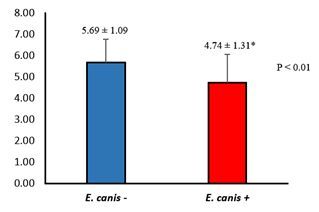
Analysis of variance (Student’s t-test) of the blood viscosity parameter of dogs in the control group (blue) and dogs with Canine Monocytic Ehrlichiosis (red). * Significant differences between groups (P values ≤ 0.05).

**Figure 2:**
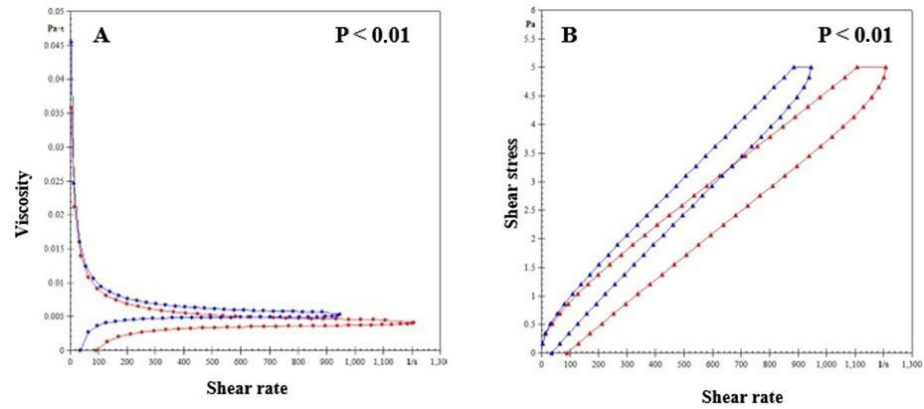
(A). Curve showing the relationship between blood viscosity variables and shear stress with shear rate in dogs with Canine Monocytic Ehrlichiosis (in red) and the control group (in blue); (B) Hysteresis areas of dogs with Monocytic Ehrlichiosis (in red) and controls (in blue). Significant differences between groups (P values < 0.01).

Dogs with *E. canis* infection (4.74 ± 0.22) had a significantly (p < 0.05) lower mean blood viscosity than the control group (5.69 ± 0.18) (Figure 1). The curves of the rheological parameters, viscosity, and shear rate are shown in Figure 2A. Both groups exhibited similar hysteresis areas (Figure 2B). The group of dogs with *E. canis* had a hysteresis area curve to the right of the graph compared to the control group.

Pearson's linear correlation analysis between rheological and hematological parameters (Table 4) demonstrated that infected dogs showed a strong and directly proportional relationship between blood viscosity and erythrocyte (r = 0.87; p < 0.05), hematocrit (r = 0.83; p < 0.05), and hemoglobin (r = 0.78; p < 0.05) parameters. The CME group also showed a directly proportional and medium-strength correlation between blood viscosity and lymphocytes (r = 0.78; p < 0.05) and triglycerides (r = 0.55; p < 0.05).

**Table 4:** Pearson's linear correlation between the hematological and rheological parameters of dogs positive for *E. canis* and controls.

	**Blood viscosity**			
	**Control**		***E. canis* (+)**	
**Hematologic parameters**	**r**	**P**	**r**	**P**
Erythrocytes	0.17	0.45	0.87	< 0.05
Hematocrit	0.14	0.53	0.83	< 0.05
Hemoglobin	0.05	0.83	0.78	< 0.05
Platelets	-0.02	0.92	0.28	0.32
Leukocytes	-0.07	0.78	0.47	0.08
Neutrophils	0.12	0.62	0.24	0.33
Lymphocytes	0.02	0.91	0.54	< 0.05
Monocytes	-0.13	0.59	0.20	0.55
Eosinophils	0.05	0.83	0.06	0.83
**Biochemical parameters**				
Total protein	-0.18	0.44	0.49	0.07
Albumin	-0.13	0.56	0.34	0.22
Globulins	-0.01	0.93	0.31	0.27
Triglycerides	0.10	0.64	0.55	< 0.05

**Caption**: (+) positive result for *E. canis.,* coefficient of correlation (r), P values ≤ 0.05 were considered statistically significant.

## Discussion

CME, a disease of global significance, induces significant hematological changes in dogs (Sainz et al., 2015). This systemic disease, characterized by mononuclear inflammation in various tissues, presents many clinical signs in dogs (Castro et al., 2022). In this study, dogs infected with *E. canis* and diverse non-specific clinical and clinical-laboratory signs are challenging to define the clinical stage of CME, particularly in animals in endemic areas (Harrus & Waner, 2011).

Dogs with CME often exhibit a range of clinical signs, including apathy, hyporexia, decreased appetite, pyrexia, and pale mucous membranes. Although these signs are non-specific, they are frequently reported in dogs with this disease and can indicate the presence of CME (Diniz & Aguiar, 2022).

CME can be difficult to diagnose because it presents in several stages and with different clinical signs (Harrus & Waner, 2011). Blood flow abnormalities and viscosity have been observed in infected dogs, and hemorheological analysis is an important method for understanding blood flow behavior in dogs with CME. After applying controlled force to blood samples, the hysteresis area is formed between an upward and a downward line, both starting at zero (Scherer et al., 2016; Silva et al., 2018). This study found that the hysteresis areas in infected dogs were similar to those in uninfected dogs. These areas appeared within the curves of the relationship between shear stress and shear rate parameters and displayed the same behavior in both groups, suggesting that the disease does not affect rheological behavior. This mechanism has also been observed in other investigations of parasitic infections, such as those caused by *Plasmodium vivax* in humans (Scherer et al., 2016), *Leishmania* sp. in dogs (Silva et al., 2018), and intracellular bacteria from the Anaplasmataceae family (Cardoso et al., 2020).

Maintaining blood flow is important to ensure the constant flow of nutrients and oxygen molecules to tissues (França et al., 2014), especially those with limited tissue storage capacity, as these components are necessary to maintain cellular life (Willie et al., 2014). This study maintained the hysteresis area even with *E. canis* infection, indicating that dogs were adapted to maintain homeostasis.

It is worth noting that this study found lower blood viscosity in dogs infected with CME. Variations in plasma or cellular components can impact the resistance to blood flow in the vascular system and tissue perfusion (Nader et al., 2019). Additionally, blood viscosity is important for maintaining the rheological properties of blood (Cabrales et al., 2007). As observed in this study, the reduced blood viscosity in dogs infected by *E. canis* has been linked to changes in hematological parameters (Cardoso et al., 2023).

In this study, the most common clinical laboratory findings in dogs with CME were thrombocytopenia, anemia, and lymphopenia, found in 80%, 70%, and 60% of the cases, respectively. Thrombocytopenia and anemia are frequently observed in *E. canis* infections (Aziz et al., 2022). *Ehrlichia canis* utilizes the iron in the blood serum for its survival metabolism, particularly in the acute phase. Consequently, a higher degree of bacteremia is linked to lower iron levels, resulting in anemia in dogs with CME (Bottari et al., 2016). Possible causes of anemia in dogs with CME are splenic sequestration and lysis by the complement system (type II hypersensitivity), suspension of medullary erythropoiesis (Oriá et al., 2008) and hemorrhages (Aziz et al., 2022). Thrombocytopenia can be caused by various mechanisms, including excessive platelet consumption due to endothelial lesions, destruction by immunological action, and increased splenic sequestration of platelets (Shropshire et al., 2018). Lymphopenia in dogs with CME is also recognized as a clinical and laboratory abnormality (Fonseca et al., 2013). Several days after the acute infection, as dogs transition into the subclinical phase of CME, there is a decrease in the production of cytokines that recruit lymphocytes, potentially leading to lymphopenia (Faria et al., 2011).

Our analysis revealed that anemia was a common hematological change in dogs infected with *E. canis*, as previously reported (Parashar et al., 2016). Infections that affect hematological parameters in dogs, such as infection with *Leishmania* sp. (Silva et al., 2018) or bacteria from the Anaplasmataceae family (Cardoso et al., 2020), have also been associated with a decrease in erythrocytes. In addition, dogs infected with *E. canis* may have reduced lymphocyte counts (Cardoso et al., 2023).

Some studies have also found changes in biochemical parameters in dogs with CME infection. In this study, infected dogs had lower albumin concentrations. The decrease in albumin levels may be related to the sequestration of albumin, an acute-phase protein, in inflamed tissues due to *E. canis* infection (Vieira et al., 2013). Albumin can be sequestered in tissues with edema, secondary to inflammation and increased capillary permeability, hemorrhages, low protein production due to concomitant hepatopathy, and can also be related to protein loss through the urine due to glomerulopathies (Harrus et al., 1999). Furthermore, some studies reported changes in total protein values ​​in infected dogs (Harrus et al., 1996; Lobetti et al., 2000; Aziz et al., 2022), but no changes in total protein levels were evidenced in this study.

Interestingly, this study found a correlation between blood viscosity and the CME group's erythrocyte, hematocrit, and hemoglobin parameters. When these hematological parameters decreased, the viscosity of the blood also decreased, as observed in dogs with CME. Similar correlations were found in dogs infected with *Leishmania* spp. (Silva et al., 2018) or bacteria from the Anaplasmataceae family (Cardoso et al., 2020), where a decrease in erythrocytes was directly associated with a decrease in blood viscosity.

The connection between hematocrit and hemoglobin levels and blood viscosity in dogs infected with *E. canis* may be important for understanding changes in blood flow in dogs with CME. However, these links were not observed in dogs infected with *Leishmania* sp. (Silva et al., 2018) or with agents from the Anaplasmataceae family (Cardoso et al., 2020).

This study showed correlations between viscosity biochemical parameters and hematological parameters. In dogs with CME, the total protein level was directly proportional to blood viscosity, suggesting that a reduction in total protein concentration may have reflected lower blood viscosity. These proteins can interact with hemorheological behaviors and decrease blood viscosity in dogs with bacteria from the Anaplasmataceae family (Cardoso et al., 2020).

The decrease in albumin levels may be related to the inflammatory nature of the disease (Vieira et al., 2013). The current study demonstrated a correlation between albumin levels and erythrocyte and hematocrit levels. The interaction of albumin with erythrocytes and hematocrit could affect the rheological behavior in dogs with CME (Irace et al., 2014). However, previous studies have not reported such interactions (Silva et al., 2018; Cardoso et al., 2020).

The correlation observed between triglycerides and erythrocytes, and neutrophils in the group of dogs with CME may influenced blood viscosity directly, although the mean triglyceride ​​in dogs with EMC did not show a significant difference with the control group. In humans, it is reported that high cholesterol levels and high counts of erythrocytes and platelets are associated with vascular diseases, and erythrocytes and serum lipids correlate with each other (Fessler et al., 2013). Another study shows that hypertriglyceridemia increases blood viscosity (Rosenson et al., 2002). In mice, it is reported evidence of greater production of pro-inflammatory cytokines by adipose tissue in individuals with hypertriglyceridemia, which leads to leukocytosis associated with vascular diseases (Sawant et al., 2021). By understanding these dynamics, triglycerides may influence rheological behavior in dogs with CME. Triglycerides can also contribute to inflammation, which can directly influence changes in blood viscosity, as reported in other studies (Scherer et al., 2016; Silva et al., 2018).

The data indicate that dogs infected with CME present blood composition and flow changes. Blood rheological parameters reflect changes in erythrocytes, hematocrit, and hemoglobin. Total protein and albumin levels are also linked to blood viscosity in infected dogs, suggesting a relationship between these factors. It is important to highlight that our study suggests that the reduction in blood viscosity in dogs infected with CME may also be linked to anemia, a direct result of the disease and its interaction with acute phase proteins, which could have significant implications for the treatment and management of CME in dogs.
